# Assessment of FDA-approved drugs against *Strongyloides ratti *in vitro and in vivo to identify potentially active drugs against strongyloidiasis

**DOI:** 10.1186/s13071-021-05117-2

**Published:** 2021-12-23

**Authors:** Jennifer Keiser, Cécile Häberli

**Affiliations:** 1 Department of Medical Parasitology and Infection Biology, Swiss Tropical and Public Health Institute, Socinstrasse 57, 4051 Basel, Switzerland; 2 University of Basel, 4003 Basel, Switzerland

**Keywords:** *Strongyloides ratti*, FDA library, Drug discovery, In vitro, In vivo

## Abstract

**Background:**

Infections with *Strongyloides stercoralis* belong to the most neglected helminth diseases, and research and development (R&D) efforts on novel drugs are inadequate.

**Methods:**

A commercially available library containing 1600 FDA-approved drugs was tested in vitro against *Strongyloides ratti* larvae (L3) at 100 µM. Hits (activity > 70%) were then evaluated against *S. ratti* adult worms at 10 µM. Morantel, prasterone, and levamisole were tested in the *S. ratti* rat model using dosages of 1–100 mg/kg.

**Results:**

Seventy-one of the 1600 compounds tested against *S. ratti* L3 revealed activity above 70%. Of 64 compounds which progressed into the adult screen, seven compounds achieved death of all worms (benzethonium chloride, cetylpyridinium chloride, Gentian violet, methylbenzethonium chloride, morantel citrate, ivermectin, coumaphos), and another eight compounds had activity > 70%. Excluding topical and toxic compounds, three drugs progressed into in vivo studies. Prasterone lacked activity in vivo, while treatment with 100 mg/kg morantel and levamisole cured all rats. The highest in vivo activity was observed with levamisole, yielding a median effective dose (ED_50_) of 1.1 mg/kg.

**Conclusions:**

Using a drug repurposing approach, our study identified levamisole as a potential backup drug for strongyloidiasis. Levamisole should be evaluated in exploratory clinical trials.

**Graphical Abstract:**

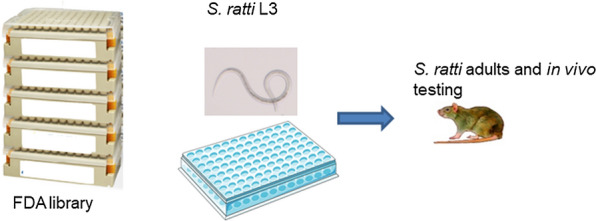

## Background

Strongyloidiasis, caused by infections with the soil-transmitted helminth *Strongyloides stercoralis* and occasionally *Strongyloides fuelleborni*, is a highly neglected tropical disease (NTD) in tropical and subtropical settings. In 2017, 614 million people were estimated to be infected, with the majority of infections occurring in South-East Asian, African, and Western Pacific regions [1]. Clinical manifestations are manifold, ranging from nonspecific gastrointestinal symptoms to severe health consequences such as hyperinfection syndromes and disseminated strongyloidiasis [2].

Ivermectin is currently the best treatment option for *S. stercoralis* infections, characterized by a high cure rate and excellent safety profile [3]. Moxidectin, also a macrocyclic lactone belonging to the milbemycin family and recently approved for onchocerciasis, shows a similar promising efficacy and safety profile [3] and would therefore qualify as novel treatment for *S. stercoralis* infection. Thiabendazole and albendazole are used as backup drugs, but have considerable limitations including lower efficacy and less favorable tolerability compared to ivermectin [4]. Additional alternative drugs are not available and not on the horizon, as there is little research and development (R&D) on drugs or diagnostics in NTDs [5, 6]. This is an alarming situation, as the widespread use of ivermectin bears a risk of the emergence of drug resistance. In the laboratory, it was shown that ivermectin resistance in *Strongyloides ratti* could be induced by the F4 generation with corresponding upregulation of some ABC isoform genes using subtherapeutic doses in rats [7]. Given the low level of funding for R&D on NTDs [5, 6], alternative low-cost strategies have been explored to increase the pool of anthelmintic drugs, including drug repurposing [8, 9].

The aim of the present study was to evaluate the activity of 1600 Food and Drug Administration (FDA)-approved drugs against *S. ratti* in an attempt to identify an alternative drug against *Strongyloides* spp. All compounds were in an initial screen evaluated against the L3 larval stage in vitro. Active compounds were followed up first against adult worms in vitro, and promising compounds were tested in vivo.

## Methods

### Drugs and media

The FDA Pharmakon compound library was purchased from MicroSource Discovery Systems, Inc. (USA). Compounds were delivered in microplates (10 mM, dissolved in DMSO) and kept at −80 °C until use. For in vivo studies, morantel, levamisole, and prasterone (DHEA) were purchased from Sigma-Aldrich (Buchs, Switzerland).

Medium RPMI 1640 was purchased from Gibco/Thermo Fisher (Waltham, MA USA), and penicillin (100 U/ml), streptomycin (100 µg/ml), and inactivated fetal calf serum (iFCS) were purchased from BioConcept (Allschwil, Switzerland).

### Animals and parasites

Three-week-old male Wistar rats (*n* = 36) were purchased from Janvier (Le Genest-Saint-Isle, France)*.* Rats were kept for 1 week in the animal facility with food and tap water ad libitum (22 °C, 50% humidity, with a 12-h light/dark cycle, 6 a.m. to 6 p.m.) before the start of the experiments.

### In vitro studies

*Strongyloides ratti* third-stage larvae (L3) were obtained from an established in-house life-cycle, as described by Garcia et al. [10]. For the drug assay, 30–40 L3 were placed in each well of a 96-well plate for each compound. Larvae were incubated in 175 µl culture medium with the test drugs at 100 µM in the dark and at room temperature for 72 h. For the readout, first, the total number of L3 per well was determined. Then, 50–80 µl of hot water (≈ 80 °C) was added to each well, and the moving larvae were counted. The proportion of larval death was determined.

Adult worms for the in vitro studies were collected from the rats’ intestines (*n* = 15 control or life-cycle rats). The intestines were opened longitudinally, washed with phosphate-buffered saline (PBS), and placed in Petri dishes containing PBS with 1% pen/strep (penicillin 100 U/ml–streptomycin 0.1 mg/ml). Plates were kept for 4 h in an incubator (37 °C, 5% CO_2_) (Innova CO-48, New Brunswick Scientific). Afterwards, the worms were collected, washed, and transferred to Petri dishes with medium RPMI 1640 (supplemented with 1% pen/strep and 5% FCS). The in vitro assays were prepared in duplicates in 24-well plates with 5–8 worms per well. The worms were incubated for 72 h in medium and tested in concentrations of 100 µM and 10 µM, and active compounds were also tested at two lower concentrations of 1 µM and 0.1 µM. Worms incubated in medium containing 0.5% DMSO served as controls. For evaluating the assays using a bright-field inverted microscope (Carl Zeiss Oberkochen, Germany, magnification ×4 and ×10), 200 µl of 80 °C hot water was added to each well to stimulate worm motility. A scale ranging from 0 to 3 was used, where 0 corresponds to dead worms (no motion within 5″) and 3 indicates maximal motility. Half maximal inhibitory concentration (IC_50_) values were calculated using CalcuSyn version 2.0 (Biosoft, Cambridge, UK).

### In vivo studies

Rats were infected orally with 800 *S. ratti* L3. Eight days after infection, rats were randomly assigned and treated with the three test drugs at 100 mg/kg. In a second experiment, morantel and levamisole were also evaluated at a single oral dose of 10 mg/kg and 1 mg/kg (levamisole only). For the oral administration, the drugs were first dissolved in a mixture of 70% Tween 80 (Sigma-Aldrich, Buchs, Switzerland) and 30% ethanol (Merck, Darmstadt, Germany), corresponding to 10% of the final volume, and then tap water was added under constant agitation using a magnetic stirrer to reach the necessary volume. Four untreated animals served as control in each of the two experiments. Seven days after administration, rats were euthanized in CO_2_, and the hosting worms were counted following the procedures described for the in vitro studies.

## Results and discussion

**Fig. 1:**
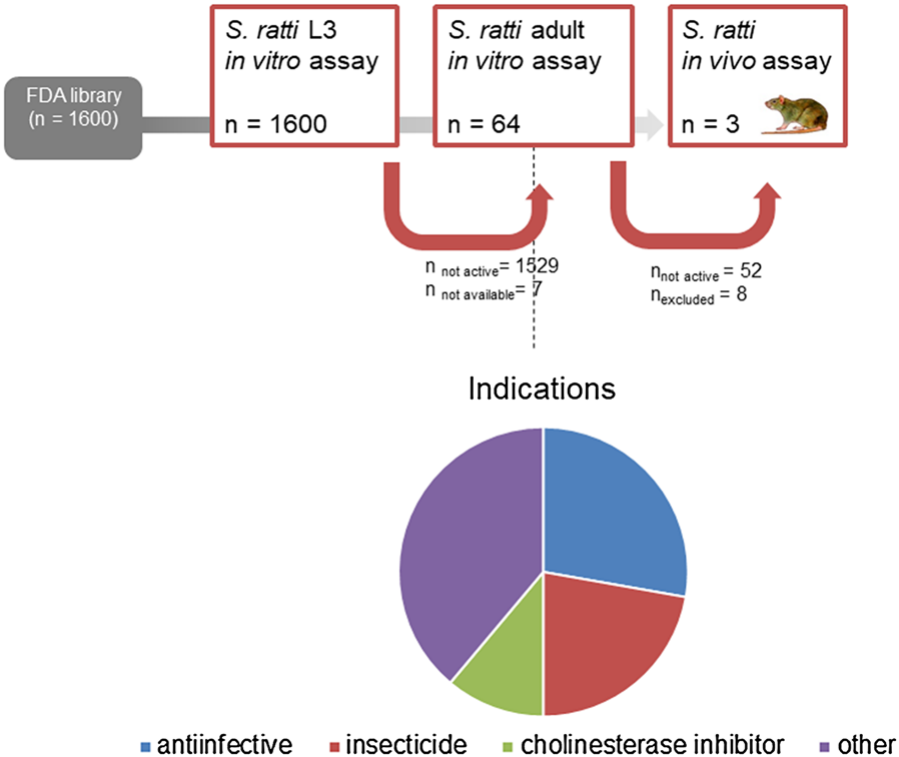
Screening flow, testing 1600 FDA compounds against *S. ratti*

Of the 1600 compounds tested against *S. ratti* L3, 71 compounds were active (defined as activity > 70%) (hit rate of 4.4%) (Fig. 1, Table 1). A threshold of 70% was applied to define activity; hence, both highly active and moderately active compounds progressed further in our screen.

**Table 1:** In vitro activity of hits against *S. ratti* L3 and adults

Drug	Effect on L3 (% dead after 72 h), [SD (%)] at 100 µM	Effect on adult worms (% dead after 72 h), [SD (%)] at 10 µM	Drug indication^d^
Aconitine	88.1 (7.5)	17.4 (5.1)	Anesthetic (gastric), antipyretic, and cardiotoxin
Acriflavinium HCl	79.4 (7.7)	97.7 (2.3)	Anti-infective, intercalating agent
Amantadine HCl	75.5 (8.2)	24.1 (4.0)	Antiviral, antiparkinsonian; treatment of drug-induced extrapyramidal reactions
Aminohippuric acid	84.8 (6.3)	32.7 (10.2)	Renal function diagnosis
Benzethonium chloride	100.0 (0)	100.0 (0)	Anti-infective (topical)
Bitoscanate	100.0 (0)	16.5 (6.4)	Anthelmintic
Brinzolamide	82.7 (4.0)	20.1 (11.4)	Antiglaucoma
Captamine	91.0 (4.6)	35.1 (9.9)	Depigmenting agent
Cetylpyridinium chloride	100.0 (0)	100.0 (0)	Anti-infective (topical)
Ceftriaxone sodium trihydrate	100.0 (0)	20.6 (11.9)	Antibacterial
Cetrimonium bromide	100.0 (0)	77.3 (11.4)	Anti-infective
Chlormidazole	93.3 (5.1)	88.6 (11.4)	Antifungal
Chloroxylenol	88.3 (2.2)	64.6 (35.4)	Antibacterial, topical, and urinary antiseptic
Chlorpyrifos	100.0 (0)	73.1 (18.8)	Insecticide
Cinnarizine	94.7 (1.0)	59.6 (40.06)	H1 antihistamine
Coumaphos	100.0 (0)	100.0 (0)	Insecticide, cholinesterase inhibitor
Cyproheptadine HCl	100.0 (0)	31.3 (3.7)	H1-antihistamine, antipruritic
Dactinomycin	71.4 (6.4)	41.7 (10.4)	Antineoplastic, intercalating agent
Debrisoquin sulfate	91.3 (1.5)	8.85 (1.3)	Anti-hypertensive
Demeclocycline HCl	81.5 (4.0)	–	Antibacterial
Dyclonine HCl	100.0 (0)	29.6 (9.5)	Anesthetic (topical)
Dibenzothiophene	90.8 (2.8)	13.2 (6.9)	Keratolytic
Dimpylate	100.0 (0)	85.6 (15.0)	Insecticide, cholinesterase inhibitor
Ebselen	96.6 (1.9)	52.5 (13.4)	Antioxidant, lipoxygenase inhibitor, inhibits oxidation of LDL
Edoxudine	89.8 (3.7)	27.2 (7.1)	Antiviral
Enilconazole sulfate	71.0 (7.9)	21.9 (3.1)	Antifungal
Enoxacin	85.4 (2.9)	44.6 (9.6)	Antibacterial
Ethisterone	100.0 (0)	52.4 (24.6)	Progestogen
Fenthion	100.0 (0)	87.0 (11.3)	Insecticide, ectoparasiticide
Floxuridine	88.9 (2.1)	62.6 (37.5)	Antineoplastic, antimetabolite
Gallamine triethiodide	88.5 (2.9)	43.1 (11.3)	Muscle relaxant (skeletal)
Gentian violet	100.0 (0)	100.0 (0)	Antibacterial, anthelmintic
Gramicidin^b^	72.7 (2.0)	32.9 (10.1)	Antibacterial
Halcinonide	96.1 (0.9)	38.7 (4.3)	Glucocorticoid, anti-inflammatory
Hexylresorcinol	83.6 (3.2)	28.8 (8.7)	Anthelmintic, topical antiseptic
Hycanthone	78.0 (15.0)	29.3 (13.5)	Anthelmintic, hepatotoxic
Hydrocortisone butyrate	77. 8 (4.0)	54.3 (20.4)	Glucocorticoid, anti-inflammatory
Imiquimod HCl	80.6 (9.2)	64.4 (24.3)	Immunomodulator
Indapamide	70.1 (6.0)	36.6 (16.5)	Diuretic, antihypertensive
Inositol	75.9 (4.1)	28.9 (8.9)	Growth factor
Iodoquinol	70.8 (5.1)	17.9 (2.2)	Anti-amoebic
Isoxicam	89.5 (2.8)	19.3 (3.6)	Anti-inflammatory
Ivermectin	100.0 (0)	8.9 (1.25)	Antiparasitic
Kanamycin A sulfate	71.0 (3.1)	50.9 (7.0)	Antibacterial
Labetalol HCl	79.9 (8.3)	41.0 (13.2)	Adrenergic blocker
Lasalocid sodium	88.7 (1.6)	10.9 (12.0)	Antibacterial
Levamisole HCl	93.1 (4.5)	88.6 (11.4)	Immunomodulatory, anthelmintic
Malathion	100.0 (0)	–	Pediculicide, insecticide, cholinesterase inhibitor
Megestrol acetate	73.3 (5.54)	51.4 (2.92)	Progestogen, antineoplastic
Methylbenzethonium chloride	91.5 (1.4)	100.0 (0)^c^	Anti-infective
Methylthiouracil	100.0 (0)	34.3 (14.2)	Antithyroid agent
Mianserin HCl	100.0 (0)	19.6 (6.9)	5HT antagonist
Mitoxantrone HCl	92.6 (9.8)	39.8 (3.1)	Antineoplastic
Morantel citrate	100.0 (0)	100.0 (0)	Anthelmintic
Nadolol	84.6 (5.8)	52.5 (17.5)	Beta-adrenergic blocker
Niclosamide	76.3 (15.2)	–	Anthelmintic, teniacide
Norethynodrel	100.0 (0)	36.8 (5.3)	Progestogen, in combination with estrogen as oral contraceptive
Oxethazaine	82.9 (6.34)	–	Anesthetic (local)
Phenothiazine	93.3 (0.8)	37.1 (2.2)	Anthelmintic
Prasterone acetate	88.6 (7.7)	77.1 (22.8)	Adrenocortical hormone, antidepressant
Prednicarbate	100.0 (0)	29.9 (13.2)	Anti-inflammatory, glucocorticoid
Prilocaine HCl	88.5 (2.2)	25.1 (5.1)	Anesthetic (local)
Proadifen HCl	74.9 (4.8)	21.3 (1.2)	Cytochrome P450 inhibitor, Ca antagonist, anesthetic (local)
Propiolactone	92.8 (2.0)	49.5 (4.81)	Anti-infective
Pyrantel pamoate	100.0 (0)	53.0 (32.9)	Anthelmintic
		52.9 (3.9)^a^	
Selamectin	79.9 (8.1)	–	Anthelmintic, antiparasitic, anti-mite
Sulfanitran	100.0 (0)	65.7 (24.8)^c^	Antibacterial, coccidiostat
Terpene hydrate	70.5 (16.6)	18.4 (4.5)	Expectorant
Tetroquinone	85.4 (4.9)	42.9 (11.4)^c^	Keratolytic
Triflupromazine HCl	90.6 (5.6)	–	Antipsychotic
Tylopaxol	84.1 (8.3)	43.8 (11.2)	Polymeric nonionic detergent

Hits are defined as compounds with 70% activity against *S. ratti* L3

^a^Considering salt factor (29 µM), empty cells reflect missing drug

^b^Gramicidin A (87%), B (7%), C (5%), and D (1%) ex *Bacillus brevis*

^c^Only tested once. SD was calculated between individual wells

^d^Indication according to library provider (Pharmakon)

The active compounds are from several therapeutic areas, including many anti-infective (and anthelmintic) agents and insecticides (Fig. 1, Table 1). Twenty-one compounds killed all *S. ratti* larvae after 72 h of incubation at 100 µM.

Sixty-four compounds (seven compounds were not available) were tested against adult *S. ratti* at 10 µM. Seven compounds achieved death of all worms (benzethonium chloride, cetylpyridinium chloride, Gentian violet, methylbenzethonium chloride, morantel citrate, ivermectin, coumaphos), and another eight compounds had activity > 70%. It is interesting to note that pyrantel pamoate was only moderately active (53%) while morantel showed high activity (100%) at 10 µM of the active ingredients. Since several of the active compounds are for topical use only (e.g., Gentian violet or cetrimonium bromide) (see Table 1) or are rather toxic (e.g., coumaphos or sodium nitroprusside), only three of the 14 compounds, namely levamisole, morantel, and prasterone, were selected for IC_50_ determination and in vivo studies. The activity of ivermectin in in vivo studies was presented in earlier work [11, 12].

Levamisole is an anthelmintic which has been tested in different veterinary formulations in ruminants, with moderate activity against *Strongyloides papillosus* in cattle [13] and *Strongyloides* spp. in goats [14]. An injectable formulation of levamisole was effective against *Strongyloides venezuelensis* in rats. A case report from a human infection documented a positive response [15]. However, a thorough evaluation of levamisole against *Strongyloides* spp. has not been done in either the laboratory or in human studies. The drug is misused as an adulterant and cutting agent in cocaine distribution [16]. Levamisole-adulterated cocaine use has been reported to cause severe adverse events including neutropenia and agranulocytosis, vasculitis, skin necrosis, and arthralgia, which resulted in withdrawal of levamisole as a human drug [17]. The drug was, however, found safe and well tolerated in the doses used as an anthelmintic in human clinical trials [18] and is still listed on the WHO list of essential list of medicines as anthelmintic (https://list.essentialmeds.org/). Levamisole is also receiving increasing attention as therapy for relapsing nephrotic syndrome [19].

Only a single study was found in the literature using morantel, which is a tetrahydropyrimidine like pyrantel and oxantel, against *Strongyloides* spp., which evaluated its activity in sheep [20]. Morantel is not used in human medicine. Lastly, prasterone is a naturally occurring androstane steroid used for menopausal symptoms [21].

The calculated IC_50_ values for levamisole, morantel, and prasterone against adult *S. ratti* at 72 h were 0.1, > 1, and 3.3 µM, respectively. The activity of levamisole against larval and adult stages was also studied at earlier time points (24 and 48 h), and the findings are presented in Table 2. Activity was clearly visible at the 24-h evaluation time point, in particular at the highest concentrations tested, and both larval and adult stages were highly affected after 48 h of incubation. The calculated IC_50_ values for levamisole against larval *S. ratti* were 2.0, 0.2, and 0.1 µM at 24, 48, and 72 h, respectively. IC_50_ values against adult worms were > 100, 0.4, and 0.1 µM at 24, 48, and 72 h, respectively.

**Table 2:** IC_50_ values of levamisole against larval and adult *S. ratti* at 24, 48, and 72 h

Developmental stage	Evaluation time point (h)	Concentration tested (µM)				
		100	10	1	0.1	IC_50_ value
L3	24	79.5 (8.4)	73.3 (4.5)	55.2 (25.2)	14.2 (1)	2.05
	48	94 8 (2.5)	92.7 (2.9)	90.3 (3.0)	16.8 (0.7)	0.25
	72	98.4 (2.8)	97.1 (2.6)	91.7 (4.4)	28.45 (2.8)	0.12
Adult	24	39.1 (2.3)	33.1 (2.1)	30.8 (1.9)	20.4 (5.6)	> 100
	48	87.5 (4.5)	84.6 (2.3)	75.0 (3.1)	25.5 (0)	0.35
	72	92.7 (2.2)	82.8 (1.4)	76.8 (2.3)	43.9 (3.5)	0.11

The in vivo results are summarized in Table 3. At 100 mg/kg, morantel and levamisole cured all rats, while prasterone was not active (worm burden reduction of 8%). At a lower dose of 10 mg/kg, worm burden reductions of 45% were obtained for morantel, while levamisole still cured all rats. At 1 mg/kg, levamisole achieved a worm burden reduction of 44%.

**Table 3:** Activity of levamisole, morantel, and prasterone in the *S. ratti* model

Treatment	Dose (mg/kg)	No. of rats cured^a^/investigated	Mean adult worm burden (SD)	Total adult worm burden reduction (%)
Control 1	No treatment	0/4	148 (13.0)	–
Control 2	No treatment	0/4	221.25 (81.5)	–
Control 3	No treatment	0/4	121.3 (15.4)	–
Levamisole	100^1^	4/4	0 (0)	100
	10^2^	4/4	0 (0)	100
	1^3^	4/4	68 (18.5)	43.9
Morantel	100^1^	3/3	0 (0)	100
	10^2^	0/4	66 (8.6)	45.2
Prasterone	100^1^	0/4	137 (39)	7.6

Superscripts refer to the respective control batch used in the experiment

## Conclusions

The use of a drug repurposing approach, screening a large library of 1600 approved drugs in vitro followed by in vivo studies, enabled the identification of levamisole and morantel as alternative drug candidates for strongyloidiasis. Since morantel is not approved for human use, a long and costly preclinical and clinical development process would be required. A shorter pathway would be foreseen for levamisole. Exploratory clinical trials are necessary to evaluate whether levamisole could serve as a backup drug in case of treatment failures with ivermectin and moxidectin.

## Data Availability

The data supporting the conclusions of this article are included within the article. Raw data are available upon request from the corresponding author.

## Abbreviation

DMSO: Dimethyl sulfoxide
